# The CLAVATA3/ESR-related peptide family in the biofuel crop pennycress

**DOI:** 10.3389/fpls.2023.1240342

**Published:** 2023-08-04

**Authors:** Lynne Hagelthorn, Jennifer C. Fletcher

**Affiliations:** ^1^ Department of Plant and Microbial Biology, University of California, Berkeley, Berkeley, CA, United States; ^2^ Plant Gene Expression Center, United States Department of Agriculture-Agricultural Research Service, Albany, CA, United States

**Keywords:** pennycress, phylogenetics, expression, CLE, development, biofuel

## Abstract

CLAVATA3/ESR-related (CLE) peptides perform a variety of important functions in plant development and historically have been targeted during the domestication of existing crops. Pennycress (*Thlaspi arvense*) is an emerging biofuel crop currently undergoing domestication that offers novel monetary and environmental incentives as a winter cover crop during an otherwise fallow period of the corn/soybean farming rotation. Here we report the characterization of the *CLE* gene family in pennycress through homology comparison of the CLE motif with other dicot species by conducting a homology comparison and maximum likelihood phylogenetic analysis supplemented with manual annotation. Twenty-seven pennycress *CLE* genes were identified, and their expression analyzed through transcriptome profiling and RT-qPCR. Our study provides a genome-wide analysis of the *CLE* gene family in pennycress and carries significant value for accelerating the domestication of this crop through identification of potential key developmental regulatory genes.

## 1 Introduction

*CLE* genes encode a family of extracellular signaling peptides that are involved in numerous plant developmental processes. *CLAVATA3 (CLV3)* is the founding member of the *CLE* gene family in *Arabidopsis thaliana* and is a primary player in maintaining the shoot apical meristem: the pool of stem cells in the shoot tip that acts as a source of all cells for above ground organ development (Fletcher et al., 1999; Beauzamy et al., 2015; Fletcher, 2018). CLV3 activity as a key stem cell regulator comprises an ancient land plant function found in plants as early as bryophytes such as *Physcomitrella patens* (Whitewoods et al., 2018). CLV3 has an overlapping function in limiting shoot meristem maintenance with CLE16 and CLE17 (Dao et al., 2022) but acts oppositely to CLE40 (Schlegel et al., 2021). Additionally, the CLE19, CLE22 and CLE40 peptides each function in the root apical meristem, with CLE19 regulating root meristem size, CLE22 playing a role in meristem maintenance, and CLE40 regulating the distance of the quiescent center from the root tip (Casamitjana-Martıínez et al., 2003; Fiers et al., 2004; Stahl et al., 2009; Jun et al., 2010). CLE41 and CLE44 are involved in determining the division plane of cambium cells in the vascular root meristem (Ito et al., 2006; Hirakawa et al., 2008; Etchells and Turner, 2010). CLE1, CLE3, CLE4 and CLE7 are implicated in lateral root development under nitrogen deficiency (Araya et al., 2014). CLE8 is involved in seed development (Fiume and Fletcher, 2012) whereas CLE9/10 and CLE25 regulate stomate and vascular bundle development (Qian et al., 2018; Song et al., 2021). Further, CLE45 activity is implicated in phloem development (Song et al., 2021).

*CLE* genes are highly conserved in plants (Oelkers et al., 2008; Zhang et al., 2020). The full-length proteins comprise a conserved structure consisting of a signal peptide sequence near the amino-terminus of the peptide, followed by a variable domain that shows relatively low amino acid similarity between CLE proteins in a given plant species, and a CLE motif towards the carboxyl-terminus (Cock and McCormick, 2001; Strabala, 2008). The CLE motif is cleaved from the full-length protein and undergoes downstream post-translational modification to generate the mature, functional 12-13 amino acid peptide (Kim et al., 2017). The structure of CLE peptides among land plants is well conserved (Oelkers et al., 2008; Zhang et al., 2020), allowing for comprehensive phylogenetic comparison between Arabidopsis and other angiosperms.

The broad range of roles the CLE peptides play reflects potential uses in future domestication; for instance, meristem enlargement can lead to increased fruit size and seed number in crops such as maize, rice and tomato (Somssich et al., 2016). Domestication of crops such as tomato and maize was undertaken through significant changes in plant architecture traits that are mediated by shoot and floral meristem size such as flower number, fruit size, and fruit number. With meristem size being primarily regulated by the *CLV3* pathway (Doebley et al., 2006; Bennetzen and Hake, 2009; Xu et al., 2015; Wu et al., 2018), understanding of the CLE family in new species can provide insight into its plant architecture and thereby provide a next step in its domestication effort by improving yield.

The midwestern United States primarily uses a rotation of corn and soybean as agricultural output. Fertilizer applied to these crops is subject to runoff into local waterways during the fallow winter period in between crop rotations; further, soil erodes from the empty fields. Cover crops can absorb excess nitrogen, preserve soil health, and offer a significant monetary incentive to growers after their primary harvest: providing an alternative to fallow winter fields (Isbell, 2009). Pennycress, a brassica closely related to Arabidopsis, has a short enough life cycle to be planted during this otherwise fallow period (Moser et al., 2009; Franzke et al., 2011; Sedbrook et al., 2014). Further, pennycress is cold hardy and its seeds can be harvested for oil (Warwick et al., 2002; Moser et al., 2009; Fan et al., 2013). Current efforts to promote pennycress as a cover crop have made it a candidate for use in the emerging biofuels industry (Phippen and Phippen, 2012). Pennycress is currently being implemented as an off-season rotation crop in the Midwest (Phippen et al., 2022) with previously generated varieties featuring various fatty acid profiles for biodiesel, jet fuel, and industrial fuel applications (Isbell et al., 2015; Esfahanian et al., 2021; Jarvis et al., 2021). As well, classic domestication traits such as early flowering and loss of seed shattering have also been engineered in pennycress to fit its planting into maize/soybean rotations while reducing loss of seed yield (Chopra et al., 2020).

We have undertaken a genome-wide analysis of the *CLE* gene family in pennycress to gain insight into their potential conservation in this emerging crop species and identify candidate genes for domestication efforts. We identify 27 *CLE (TaCLE)* genes in the pennycress genome. These genes are highly conserved between pennycress and Arabidopsis, although several sets of homologous *CLE* gene pairs in Arabidopsis are present as single copy genes in pennycress. Transcript profiling using various pennycress tissues shows that *TaCLE* genes are expressed in a variety of tissues during plant development; as well, some pennycress *CLE* genes show tissue expression profiles distinct from their Arabidopsis counterparts. Defining the pennycress CLE family members provides candidates for genetic engineering that can be undertaken to accelerate the domestication of this emerging biofuel crop.

## 2 Methods

### 2.1 Hidden markov modelling and sequence identification

HMMer 3.3.2 was used to identify amino acid sequences containing a CLE motif in the pennycress genome annotation version 1 (Dorn et al., 2015) using the hmmscan command and a CLE profile.hmm file generated by Oelkers et al., 2008. nblastn searches of the annotated genomic sequences of each of the 32 Arabidopsis *CLE* genes were then used to further identify and refine candidate sequences found during the HMM search. Finally, manual comparison of pennycress candidate peptides with Arabidopsis peptides and Sanger sequencing of pennycress cDNA amplified from whole seedling tissue using predicted *TaCLE* gene-specific primers (**Supplementary Table 2**) provided final consensus on identified sequences.

### 2.2 Multiple sequence alignment and conserved motif analysis

Multiple sequence alignment was performed on pennycress peptides using Clustal Omega and visualized using Jalview (Waterhouse et al., 2009; Madeira et al., 2022). Visualization of the 27 pennycress and 32 Arabidopsis peptide consensus sequences was undertaken using the publicly available weblogo application (Crooks et al., 2004) using default settings.

### 2.3 cDNA extraction and sequencing

MN106 seeds were sown on MS-Agar plates and seedlings were allowed to grow under long day (16 hour light: 8 hour dark) conditions at 22°C for three weeks after germination (Murashige and Skoog, 1962). RNA was extracted from 10 whole seedlings using a commercial RNeasy mini kit (Qiagen, 74004). Application of DNase I was used to destroy remaining genomic DNA according to the manufacturer’s protocol (Thermo Fisher Scientific, M0303S). 1 µg of extracted RNA was reverse transcribed using a commercial cDNA synthesis kit (Bio-Rad, 1708890). *CLE* gene sequences were amplified from 1 µl of 1/20^th^ dilution of 1000 ng/µl MN106 cDNA using gene-specific primers (**Supplementary Table 2**) and the resulting DNA amplicons run on gels and extracted using the QIAquick gel extraction kit (Qiagen, 28706). Extracted DNA fragments were sequenced through Eurofins Genomics (Eurofins Genomics LLC).

### 2.4 Phylogenetic comparison and genomic organization

*CLE* genomic nucleotide sequences were aligned using Clustal Omega and the terminal ends were eliminated in Jalview alignment to align the signal peptide sequences as well as CLE motifs (Waterhouse et al., 2009; Madeira et al., 2022). One thousand phylogenetic maximum likelihood trees were generated using RAxML to generate sufficient bootstrap values and visualized using iTOL (Kozlov et al., 2019; Letunic and Bork, 2021). The following command was issued for RAxML generation:

raxmlHPC-PTHREADS-SSE3 -f a -x 1123 -p 2341 -#1000 -m GTRGAMMA -T 3 -s [Alignment File].fa -n [Output File].raxml

Genomic organization was visualized using the gene structure display server (Hu et al., 2015). Signal peptide analysis of full-length TaCLE amino acid sequences was undertaken using the SignalP web browser on the ‘Eukarya’ setting (Teufel et al., 2022).

### 2.5 Expression analysis

*In silico* transcription analysis was undertaken using a publicly available transcriptome dataset (Dorn et al., 2013) to generate a heatmap using ggplot (Wickham, 2016). Individual *TaCLE* gene expression within nine tissue types was profiled from a publicly available dataset (Nunn et al., 2022), using a minimum normalized read count cutoff of 10 or higher. For RT-qPCR, MN106 seedlings were grown on ½ MS-Agar plates and harvested three weeks after germination for RNA extraction using the Qiagen RNeasy Mini Kit. Genomic DNA was digested using NEB DNase I and the remaining RNA was reverse transcribed using the Bio-Rad iScript Reverse Transcription Supermix. Finally, qPCR was undertaken using the Bio-Rad Syber Green Master Mix. Differential expression analysis was performed manually taking Cq values according to the following formulae:

ΔCq = Cq_Target_ – Cq_Actin_

ΔCq Expression = 2^–ΔCq^

and normalizing the ΔCq of the different tissue types to that of an *ACTIN* reference gene. A cutoff of a p-value < 0.05 from a one-tailed student’s t-test between null and experimental values was used to establish detectable expression. Three technical replicates were performed for each of three biological replicates.

## 3 Results

### 3.1 Identification and verification of CLE gene family in *Thlaspi arvense*

Extracting the predicted CLE peptides from the publicly available *Thlaspi arvense* version 1 genome using a downloadable FASTA file generated a searchable protein sequence list from which a Hidden Markov Model (HMM) search was conducted using HMMer 3.3.2 (Eddy, 2011; Dorn et al., 2015). Protein sequences were verified as members of the CLE family based on the presence of a conserved signaling peptide sequence as well as a characteristic CLE motif sequence (Cock and McCormick, 2001). Corresponding genomic and coding sequences were identified based on these peptides using a CLE HMM profile (Oelkers et al., 2008). Further manual characterization of pennycress *CLE* coding sequences was undertaken using tblastn of predicted pennycress CLE proteins against known Arabidopsis *CLE* genes. Pairwise sequence alignment after tblastn verified similarity of sequence and annotation of homologous coding sequence between Arabidopsis and pennycress *CLE* sequences. Finally, genomic DNA and cDNA were isolated from 3-week-old pennycress seedlings and sequenced to confirm the annotated sequences of the *Thalaspi arvense CLE* (*TaCLE)* genes. This work yielded 27 identified *TaCLE* genes as well as their respective genome annotation location on the pennycress chromosome scaffolds (**Table 1**). Pennycress *CLE* genes were named based on their overall amino acid sequence similarity to the corresponding Arabidopsis *CLE* gene. When a single *TaCLE* gene showed strong similarity to two Arabidopsis *CLE* genes across the full coding sequence, the *TaCLE* gene was named according to which Arabidopsis *CLE* gene showed the greatest degree of similarity in the CLE domain.

**Table 1:** Complete list of *CLE* genes identified in the pennycress genome. Gene Symbol refers to the given name based on the closest sister peptide in Arabidopsis.

Gene ID	Gene Symbol	Scaffold Number	Region Start	Region End
Ta06238	TaCLE1	Ta_scaffold_14	303838	304065
Ta12956	TaCLE2	Ta_scaffold_68	108852	109088
Ta20399	TaCLE3	Ta_scaffold_215	158462	158707
Ta20396	TaCLE5	Ta_scaffold_215	127746	127928
Ta20398	TaCLE7	Ta_scaffold_215	146437	146685
Ta20781	TaCLE8	Ta_scaffold_498	48083	48442
Ta21951 Exon2	TaCLE9	Ta_scaffold_310	141476	141780
Ta06692	TaCLE10	Ta_scaffold_57	644886	645215
Ta19031	TaCLE11	Ta_scaffold_166	78300	78587
Ta06630	TaCLE12	Ta_scaffold_57	259627	259860
Ta18354	TaCLE13	Ta_scaffold_149	85468	85791
Ta16559	TaCLE14	Ta_scaffold_185	250756	251010
Ta21355	TaCLE17	Ta_scaffold_472	31610	31838
Ta03296	TaCLE18	Ta_scaffold_32	470490	470810
Ta22838	TaCLE19	Ta_scaffold_394	12206	12431
Ta05369	TaCLE20	Ta_scaffold_12	365511	365741
Ta11127	TaCLE21	Ta_scaffold_123	296187	296480
Ta05072	TaCLE22	Ta_scaffold_11	342739	343047
Ta17778	TaCLE25	Ta_scaffold_138	89678	92941
Ta24317	TaCLE26	Ta_scaffold_632	88256	88888
Ta04974	TaCLE27	Ta_scaffold_69	765453	765734
Ta05108	TaCLE40	Ta_scaffold_11	501099	501895
Ta20730	TaCLE41	Ta_scaffold_444	81656	81958
Ta06474	TaCLE42	Ta_scaffold_83	767787	768053
Ta17144	TaCLE43	Ta_scaffold_125	259011	259295
Ta19767	TaCLE45	Ta_scaffold_194	43788	44150
AtCLV3HomologousRegion	TaCLV3	Ta_scaffold_788	39741	40551

Region start and region end denote the genomic location of the coding sequence as well as the upstream and downstream untranslated regions (UTRs) on the corresponding scaffold from Pennycress Genome Annotation v1.

Sequencing of amplified cDNA using *TaCLE* gene-specific primers confirmed the corrected coding sequence annotation and generated updated scaffold locations for the *TaCLV3, TaCLE19, TaCLE25* and *TaCLE40* loci (**Table 1**). *TaCLV3* was not annotated in the Pennycress Genome Annotation v1 (Dorn et al., 2015) so we mapped it to Ta_scaffold_788 based on nucleotide sequence homology to the Arabidopsis *CLV3* gene (Fletcher et al., 1999). The genomic sequence corresponding to *TaCLE19* was originally annotated as the second exon of a larger gene with the Gene ID Ta21951. Our reannotation of the *TaCLE25* coding sequence removes 25 base pairs (bp) from the 5’ end and 1 bp from the 3’ end of exon 1, as well as 28 bp from the 3’ end of exon 2, relative to the draft genome annotation (**Figure S1**). Finally, our reannotation of *TaCLE40* identifies exon 1 as a 100 bp sequence that initiates 179 bp downstream of the exon 1 sequence annotated in the draft genome, and additionally omits 22 bp from the 3’ end of exon 2 (**Figure S1**). A recent release of an improved pennycress reference genome assembly representing ~97.5% of the estimated genome size (Nunn et al., 2022) correctly presented the sequences of the *TaCLV3* and *TaCLE40* genes, denoted TAV2_LOCUS13686 and TAV2_LOCUS22323, respectively, illustrating the limitations of the initial assembly built from shorter-read sequences.

**Figure 1:**
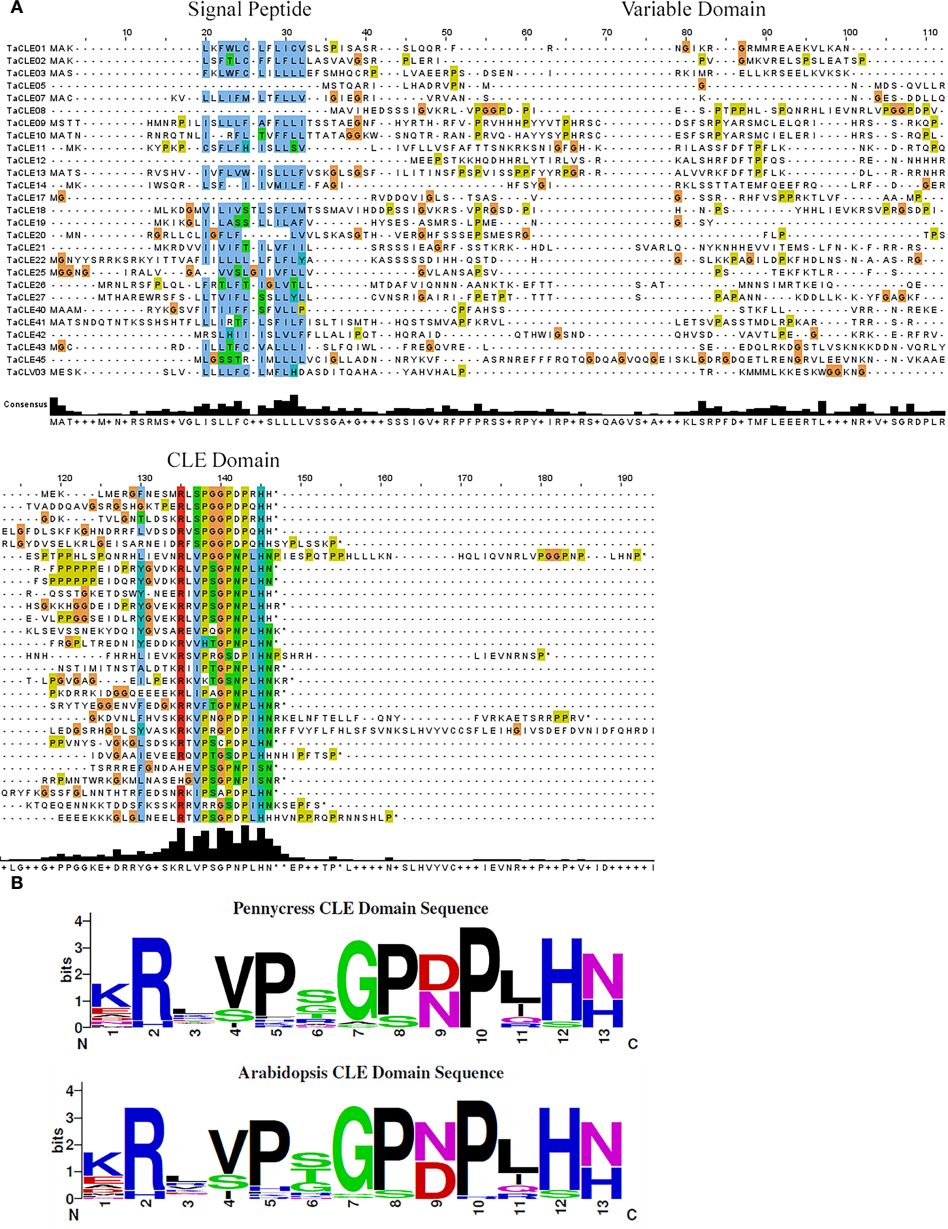
Multiple sequence alignment of the TaCLE proteins and consensus sequence for the CLE domains of pennycress and Arabidopsis. **(A)** Multiple sequence alignment of all 27 TaCLE proteins with the signal peptide, variable domain and CLE domain regions denoted. Colored boxes indicate conserved amino acids, and an amino acid consensus plot is shown below the CLE peptide sequences. **(B)** Weblogo plots of the pennycress and Arabidopsis CLE domain amino acid consensus sequences.

A multiple sequence alignment of the full-length TaCLE proteins was constructed using Clustal Omega and Jalview (Waterhouse et al., 2009) to observe the conservation of amino acid sequences among the family members. TaCLE peptides display the characteristic conserved amino-terminal signal peptide sequence as well as the highly conserved 13 amino acid CLE motif located towards the C-terminus (**Figure 1A**). Similar to the full-length Arabidopsis CLE proteins (Cock and McCormick, 2001), the intervening variable domain presents little conservation between the different full-length CLE proteins in pennycress (**Figure 1A**).

All 32 Arabidopsis CLE proteins contain an amino-terminal hydrophobic region that is predicted to act either as a signal peptide or a signal anchor sequence, directing the CLE peptide to the extracellular space (Sharma et al., 2003). Interestingly, the TaCLE5, TaCLE8, TaCLE12 and TaCLE17 proteins seem to lack a conserved signal peptide based on the amino acid sequence alignment (**Figure 1A**). We confirmed this prediction by examining their full-length amino acid sequences using SignalP 6.0, a machine learning model that detects all five known signal peptide types (Nielsen et al., 2019; Teufel et al., 2022). SignalP 6.0 failed to predict a signal peptide in any of these four pennycress CLE proteins (**Table S1**). Experimental analysis of these proteins therefore will be required to determine their subcellular localization.

The CLE domain amino acid consensus between pennycress and Arabidopsis proteins is highly similar (**Figure 1B**). The most striking difference lies at (N/D)_9_ with aspartate being slightly more prevalent in pennycress when compared to Arabidopsis. Divergence also exists at the sixth residue with a prevalence of(S/G/T)_6_ in pennycress compared to (S/T/G)_6_ in Arabidopsis; however, other sites display high levels of conservation (**Figure 1B**). CLE peptides undergo various post-translational modifications to gain functional potence (Kondo et al., 2006; Ohyama et al., 2009). Proline_8_, the site required for glycosylation, is highly conserved among the pennycress CLE domains (**Figure 1B**), which is consistent with our understanding of the importance of glycosylation at Pro_8_ of CLV3 to facilitate its ability to restrict stem cell activity (Shinohara and Matsubayashi, 2013). While experimental evidence will be necessary to assess the biochemical activities of the pennycress CLE peptides, conservation of the functional residue suggests overall conservation of function.

### 3.2 Phylogenetic analysis and genome organization of TaCLE genes

**Figure 2:**
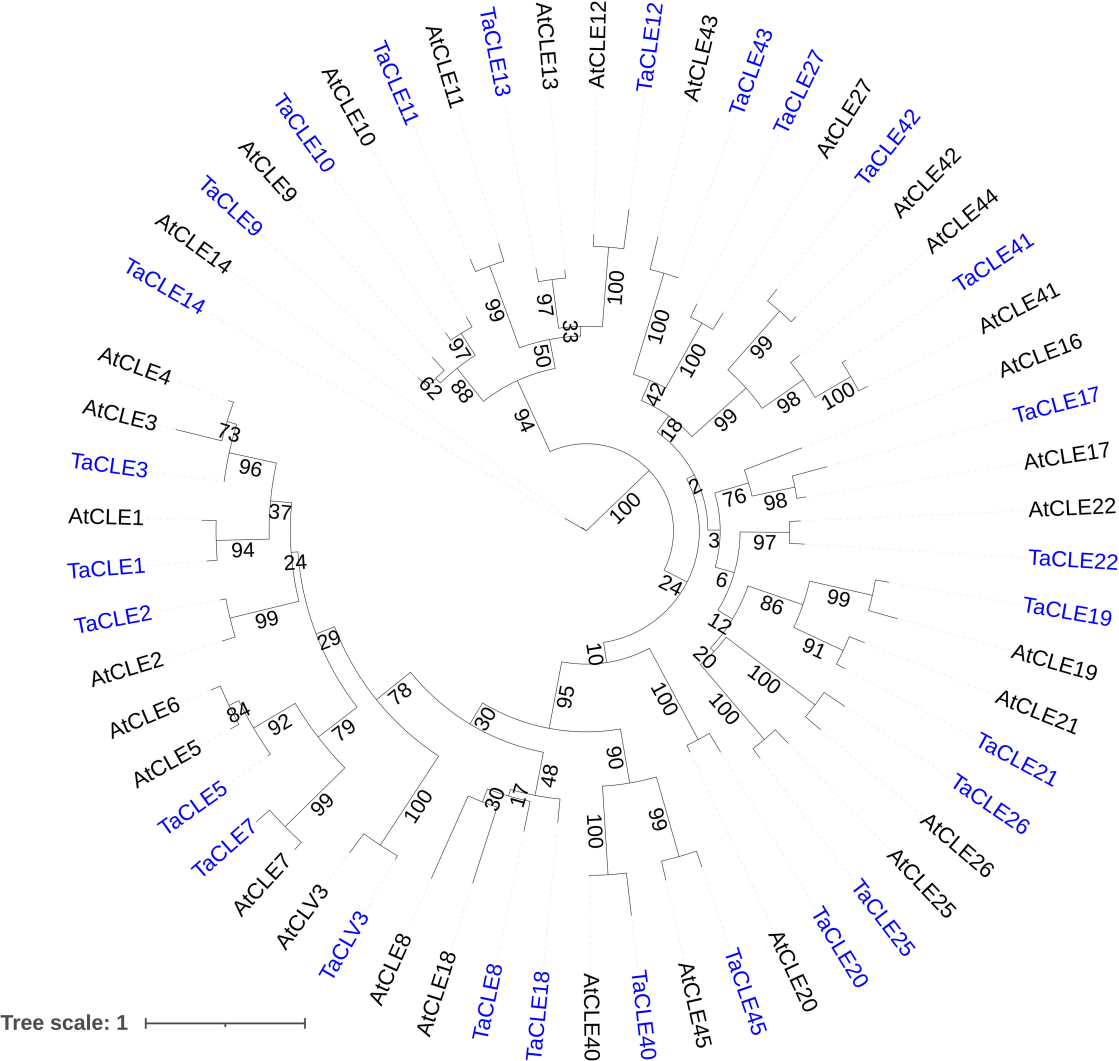
Phylogenetic analysis of pennycress and Arabidopsis CLE peptides. Maximum likelihood tree generated from alignment of all pennycress (blue) and Arabidopsis (black) CLE domains with 1000 bootstrap replicates. Bootstrap values from 1-100 are displayed at each branch.

We next determined the general nucleotide sequence similarity of the pennycress to the Arabidopsis *CLE* genes through a maximum likelihood phylogenetic analysis. A Hidden Markov Model, BLAST comparison, and manual homology modeling of genomic nucleotide sequences followed by maximum likelihood tree generation shows a near one-to-one correspondence between pennycress *CLE* genes and their relatives in Arabidopsis (**Figure 2**). An exception occurs with the *TaCLE8* and *TaCLE18* genes, which are named based on the TaCLE8 CLE motif having greatest similarity to that of AtCLE8 and the TaCLE18 CLE motif having greatest similarity to that of AtCLE18. Our phylogenetic analysis shows that the *AtCLE8* and *AtCLE18* genomic nucleotide sequences are more similar to one another than either is to the *TaCLE8* or *TaCLE18* sequences (**Figure 2**); however, due to the low bootstrap values we are unable to draw this conclusion definitively. Interestingly, the gene pairs *AtCLE3/4, AtCLE5/6, AtCLE16/17* and *AtCLE41/44* that are predicted to be the result of duplication events in Arabidopsis (Sharma et al., 2003) each have one homologue in pennycress, denoted *TaCLE3, TaCLE5, TaCLE17*, and *TaCLE41* respectively based on which of the two Arabidopsis homologues has the greatest similarity to the pennycress CLE domain (**Table 1**; **Figure 2**). Overall, the bootstrap values for the various branches of the tree are reproducibly high (>50), demonstrating that the pennycress *CLE* genes are reproducibly different from one another while maintaining a largely one-to-one relationship with their sister Arabidopsis genes.

**Figure 3:**
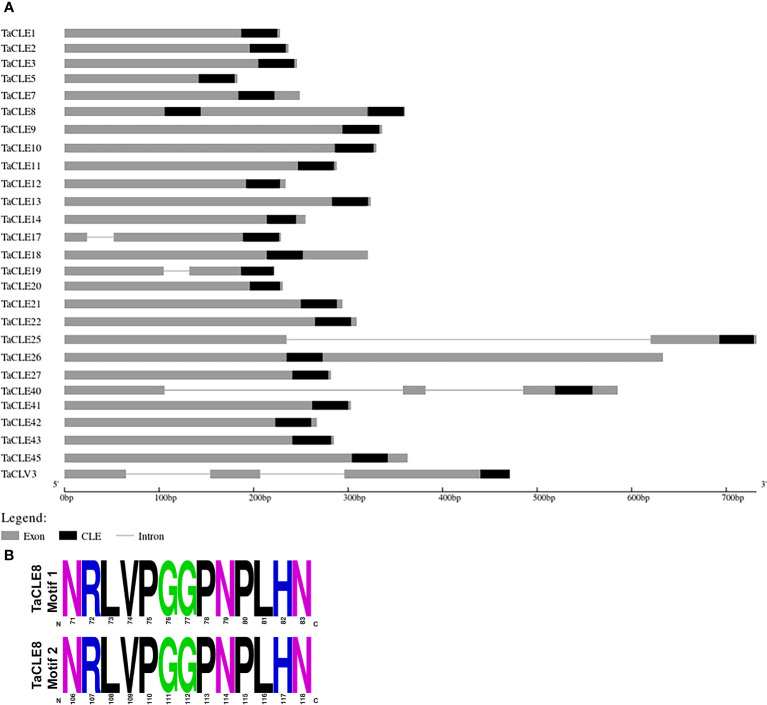
Genomic organization of the *TaCLE* genes and sequence of the duplicate TaCLE8 CLE domains. **(A)** *TaCLE* genome structure showing exons as grey boxes, introns as lines, and CLE motifs as black boxes. **(B)** Amino acid sequences of the two TaCLE8 CLE domains.

Examination of the genomic organization of the *TaCLE* loci indicates that the genes predominantly lack introns and that the length of the genomic sequence is on the order of 200-700 base pairs (**Figure 3A**). In Arabidopsis, both the *CLV3* and *CLE40* loci consist of three exons and two introns (Cock and McCormick, 2001), and these gene structures are conserved in their pennycress counterparts. Yet whereas *AtCLE16, AtCLE17*, *AtCLE19* and *AtCLE25* consist of a single exon, the *TaCLE17* and *TaCLE19* loci both feature a small intron and the *TaCLE25* locus features a much longer intron (**Figure 3A**), suggesting the regulation of these three genes may differ from that of their Arabidopsis counterparts.

Although the 32 Arabidopsis *CLE* genes each contain a single CLE motif, some *CLE* genes in other plants contain multiple CLE motifs (Gao and Guo, 2012). Among the 27 identified *CLE* genes in pennycress, the *TaCLE8* gene encodes two separate CLE motifs, one located in the center and the other towards the C terminus of the protein (**Figure 3A**). The CLE domains of TaCLE8 are identical in amino acid sequence (**Figure 3B**), suggesting they may have identical functions.

### 3.3 TaCLE gene expression analysis

Next, we analyzed the expression profiles of the pennycress *CLE* genes, as understanding gene transcription levels and patterns can provide insight into their involvement in various aspects of growth and development. We first used a publicly available transcriptome database (Dorn et al., 2013) to examine *TaCLE* gene expression in four broad tissue types: flowers, inflorescences, roots, and vegetative rosettes. Four main expression groups could be observed, which we demarcated Groups I-IV based on their differential expression patterns (**Figure S2**). The Group I genes *TaCLE5, TaCLE10*, and *TaCLE17* display relatively high transcript levels across all four tissue types when compared to the other *TaCLE* genes. Group II consists of 12 *TaCLE* genes with low or undetectable levels of expression in the four tissues. Group III features 8 genes with detectable mRNA expression in one or more of the tissues, but with far lower expression levels than either Group I or IV genes. Finally, the Group IV genes *TaCLE1* and *TaCLE11* show comparatively high expression in roots and rosettes relative to the Group I genes that display moderately high expression across all four tissue types. This analysis demonstrates that nearly half of the *TaCLE* genes are transcribed in one or more of the major pennycress tissue types.

**Figure 4:**
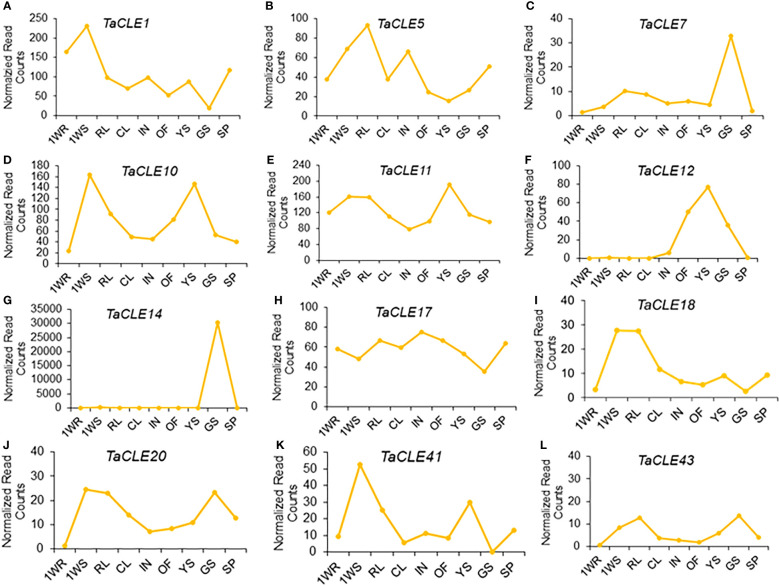
Expression profiles of *TaCLE* genes in various pennycress tissues. mRNA expression levels (normalized read counts) of the **(A)** *TaCLE1* **(B)** *TaCLE5* **(C)** *TaCLE7* **(D)** *TaCLE10* **(E)** *TaCLE11* **(F)** *TaCLE12* **(G)** *TaCLE14* **(H)** *TaCLE17* **(I)** *TaCLE18* **(J)** *TaCLE20* **(K)** *TaCLE41* and **(L)** *TaCLE43* genes. 1WR, roots from 1-week-old seedlings; 1WS, shoots from 1-week-old seedlings; RL, rosette leaves; CL, cauline leaves; IN, inflorescences; OF, open florescences; YS, young green siliques; GS, green seeds; SP, seed pods.

We further took advantage of a larger-scale expression atlas to profile *TaCLE* gene expression in nine distinct pennycress tissue types (Nunn et al., 2022). *TaCLE1* is primarily expressed in 1-week-old roots and shoots with a subsequent increase in expression in seed pods (**Figure 4A**). *TaCLE5* is primarily expressed in shoots, rosette leaves, and inflorescences (**Figure 4B**). *TaCLE7* is predominantly expressed in green seeds (**Figure 4C**) whereas *TaCLE10* and *TaCLE11* are more highly expressed in 1-week-old shoots and young green siliques (**Figures 4D, E**). *TaCLE12* is expressed in open flowers, young green siliques and green seeds (**Figure 4F**). The expression pattern of *TaCLE14* appears to be restricted to green seeds (**Figure 4G**). *TaCLE17* features broad expression, particularly in the developing rosette leaves, inflorescences and reproductive tissues (**Figure 4H**). *TaCLE18* and *TaCLE20* are primarily expressed in 1-week-old shoots and rosette leaves while *TaCLE20* is also expressed in green seeds (**Figures 4I, J**). Finally, the *CLE* genes *TaCLE41* and *TaCLE43* are both expressed in 1-week-old shoots, with also expressed in young green siliques and *TaCLE43* in green seeds (**Figures 4K, L**). Among the pennycress *CLE* genes *TaCLE1* is the most highly expressed, but others such as *TaCLE5, TaCLE7, TaCLE18, TaCLE20, TaCLE41*, and *TaCLE43* show low levels of expression (**Figure S3**). In contrast, the extremely high expression of *TaCLE14* in green seeds is likely to be an artefact of sequencing as it is orders of magnitude greater than those of the other *TaCLE* genes in either published transcriptome dataset (**Figure 4**; **Figure S2**).

**Figure 5:**
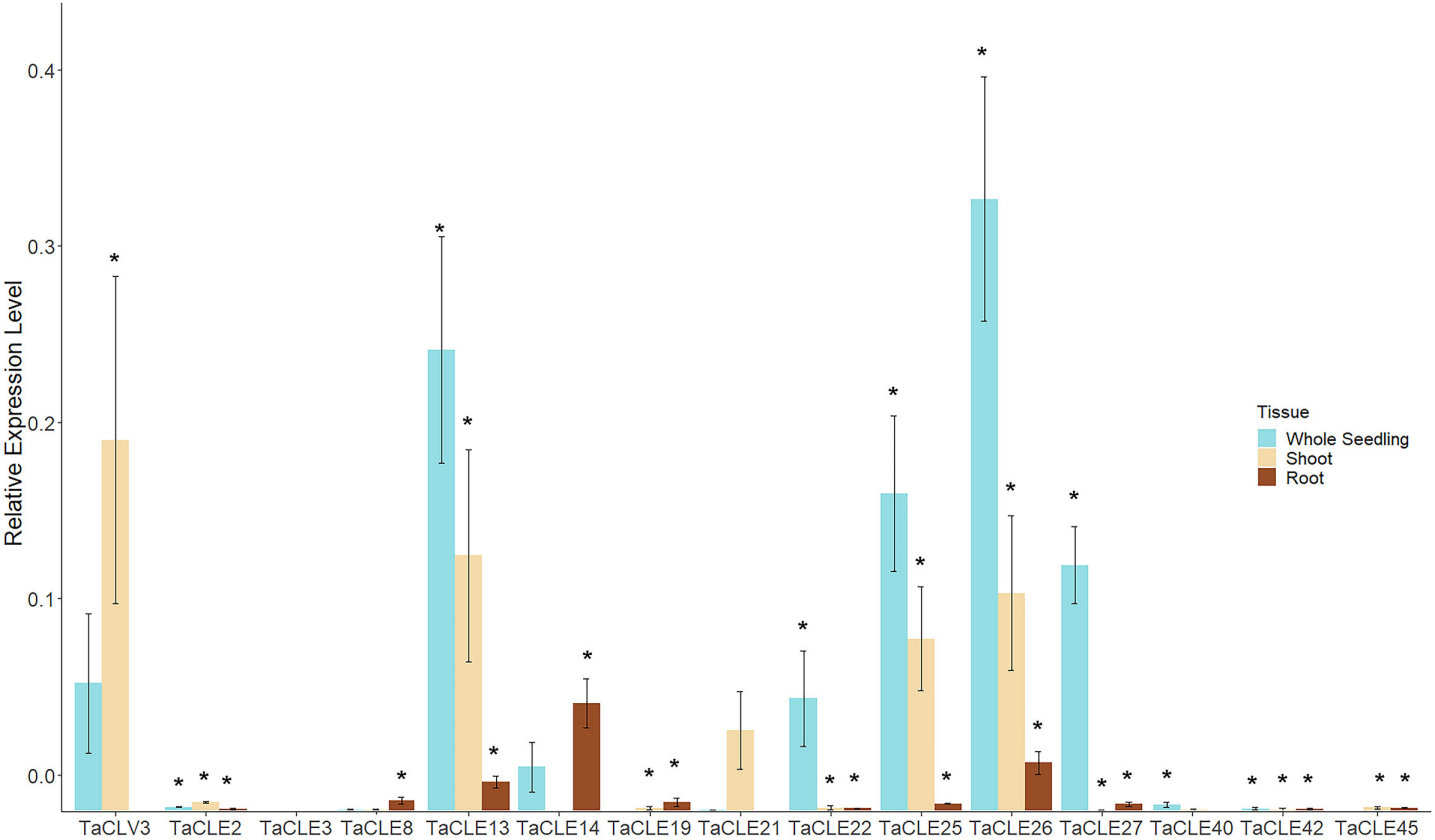
Expression profiles of *TaCLE* genes in 3-week-old pennycress tissues. Relative mRNA expression levels (mean ± SE) of *TaCLE* genes in whole seedling, shoot and root tissues. Asterisks (*) denote significance of p < 0.05 when compared via a Student’s one-tailed *t-*test against the null value.

In total, 13 out of the 27 pennycress *CLE* family members display measurable expression in the published transcriptome datasets. To elucidate the expression of the other 14 *TaCLE* genes, we queried development stages not previously examined by extracting RNA from three-week-old whole seedlings as well as dissected shoots and dissected roots, and then undertaking reverse transcription-quantitative polymerase chain reaction (RT-qPCR) to quantify *TaCLE* gene transcript levels. Most of the *CLE* genes are found to be significantly expressed using a one-tailed Student’s *t-*test against a negative control in at least one tissue type, except for *TaCLE3* and *TaCLE21* which do not feature significant levels of expression in these tissues (**Figure 5**). Importantly, quantitative PCR also confirmed the expression of our annotated versions of the *TaCLV3, TaCLE19, TaCLE25* and *TaCLE40* genes. Due to the differences between our putative sequence for *TaCLE40* and the annotated sequence in the draft genome with exon 1 being 179 base pairs apart, we designed one primer for our sequence and one for the draft sequence. Only our primer was successful in cDNA fragment amplification (**Figure S2**; **Table S2**).

Transcripts from the *TaCLE2, TaCLE19, TaCLE42* and *TaCLE45* genes are expressed at low but detectable levels in all three tissues analyzed (**Figure 5**). *TaCLV3* displays higher expression in shoot than whole seedling tissue but is barely detectable in root tissue (**Figure 5**). *TaCLE13, TaCLE25* and *TaCLE26* show similar patterns of expression, with their transcript levels highest in whole seedlings, lower in shoot tissue and very low in root tissue. *TaCLE22, TaCLE27* and *TaCLE40* transcripts are detected in whole seedling but not shoot or root tissue, suggesting they may be expressed in leaves. *TaCLE8* and *TaCLE14* appear to be expressed at low levels in root tissue, whereas *TaCLE21* transcripts are not detected at statistically significant levels in seedling, shoot or root tissues. In sum, we provide evidence for the expression under normal growth conditions of 25 of the 27 pennycress *CLE* genes.

## 4 Discussion

The *CLE* genes form an evolutionarily-conserved signaling protein family that control numerous aspects of plant growth and development. Some of these genes regulate molecular pathways that may be exploited to accelerate the domestication of orphan or emerging crops such as the biofuel crop pennycress. For example, the *CLV3*-mediated meristem maintenance pathway that has been targeted to enhance fruit and seed yield during the domestication of tomato, rice and maize (Fletcher, 2018) may be engineered to improve the low seed yield of pennycress plants (Sedbrook et al., 2014). Likewise, targeting the pennycress homolog of *CLE8* could enlarge the size of the oilseeds to improve the harvest for biofuel applications, whereas manipulation of the pennycress *CLE1-7* genes could enhance shoot regeneration and thus improve transformation efficiency (McGinn et al., 2019).

We have therefore undertaken an investigation into the CLE peptide family in pennycress through the identification of 27 *TaCLE* genes. Overall, the *TaCLE* genes display similar genetic organization and sequence composition to their Arabidopsis counterparts. We detected expression of 13 of the *TaCLE* genes in publicly available transcriptome datasets, and expression of a further 12 genes in our RT-qPCR dataset from seedling tissues. Because CLE peptides have previously been implicated in crucial developmental pathways, this study provides a solid foundation for genetic engineering of pennycress architecture, a key step in its wider adoption as a cost-effective biofuels crop.

Pennycress features a similar number of CLE peptides as other Brassica species, such as 29 in *Brassica rapa*, 32 in *Brassica oleracea*, and 32 in *Arabidopsis thaliana* (Xie et al., 2022). Full-length pennycress CLE proteins display a similar structure to CLE proteins in other species, featuring a signal peptide, variable domain, and CLE domain (**Figure 1**). Interestingly, SignalP predicts no signal peptide in TaCLE5, TaCLE8, TaCLE12 and TaCLE17, indicating either that these proteins are not exported or they lack a predictable signal peptide export sequence (**Table S1**). The TaCLE8 protein is also distinct in featuring two identical CLE domains (**Figure 3B**), indicating a possible duplication of this motif. Overall, the CLE domain consensus sequence shows greater conservation between Arabidopsis and pennycress (**Figure 1B**) than between Arabidopsis and *Brassica rapa, Brassica napus* or *Brassica oleracea* (Xie et al., 2022). This high level of sequence conservation is suggestive of conserved roles for CLE family members between Arabidopsis and pennycress.

Phylogenetic analysis and hidden Markov modelling showcase a near one-to-one correspondence between Arabidopsis and pennycress *CLE* genes, although notably the Arabidopsis gene pairs *AtCLE3/4, AtCLE5/6, AtCLE16/17*, and *AtCLE41/44* have only one copy in pennycress (**Figure 1A**; **Figure 3**). Yet *AtCLE9/10*, another predicted gene duplication in Arabidopsis, features both homologues in pennycress. These divergences suggest that either these *CLE* genes were duplicated in a common ancestor of Arabidopsis and pennycress and then subsequently some were lost in pennycress, or the genes have undergone duplication following the divergence between these two closely related Brassica species. Further phylogenetic analysis of the CLE family in closer relatives of pennycress could help to distinguish between these hypotheses.

Generally, the pennycress *CLE* gene structures are similar to those of the Arabidopsis *CLE* genes (**Figure 3A**). Most *TaCLE* loci consist of a single exon, although *TaCLE40* and *TaCLV3*, like *AtCLE40* and *AtCLV3*, consist of two introns and three exons. Yet unlike their Arabidopsis counterparts, the *TaCLE17, TaCLE19* and *TaCLE25* genes each contain a single intron, suggesting differences may exist in their transcription regulation. The overall similarity in gene structure is demonstrative of the relatedness between these two species.

To gain insight into the potential biological functions of the pennycress *CLE* genes, we examined their transcription profiles using *in silico* expression analysis as well as RT-qPCR. We found evidence for the expression of 25 *TaCLE* genes within the various tissues profiled (**Figures 4**, **5**, **S2**). However, we were unable to detect measurable *TaCLE3* and *TaCLE21* expression across the datasets sampled, and several *TaCLE* gene transcripts, including *TaCLE42, TaCLE43* and *TaCLE45* were detected at extremely low levels. This is not unexpected because many *CLE* genes in various species are expressed at very low levels, making them difficult to parse in transcriptomics data (Goad et al., 2017). Further, some *CLE* genes are restricted to small subsets of tissues: for example, *CLV3* expression is restricted to the central zone of the Arabidopsis shoot and floral meristem (Fletcher et al., 1999). These attributes can make accurately quantifying transcript levels within bulk tissues such as leaves or roots difficult. Future analysis using transcriptional reporter lines can more accurately pinpoint when and where the *TaCLE* genes are expressed.

Importantly, absolute or relative gene expression values are not necessarily reflective of the importance of a given gene in growth and development. *CLE* genes that are lowly expressed may still carry out important functions. Further work will clarify the expression of the entire *TaCLE* gene family in tissues not yet assayed, such as floral organs and lateral roots, and determine the precise gene expression patterns within tissues.

Like the Arabidopsis *CLE* genes (Jun et al., 2010), the majority of *TaCLE* genes are detected in multiple tissues during pennycress development (**Figures 4**, **5**; **Figures S2**, **S3**). In addition, all pennycress tissues examined express multiple *TaCLE* genes. We observe that a number of *CLE* genes including *TaCLE1, TaCLE5, TaCLE11*, and *TaCLE17* are broadly expressed in pennycress, whereas others such as *TaCLE12* and *TaCLE18* show more tissue-restricted expression patterns.

Many *TaCLE* genes are expressed in pennycress roots, as is the case in Arabidopsis (Jun et al., 2010). In root tissue from 1-week-old seedlings*, TaCLE1, TaCLE5, TaCLE9, TaCLE11*, and *TaCLE17* are all expressed, as are *TaCLE10* and *TaCLE41* at very low levels (**Figures 4**, **S2**). In root tissue from 3-week-old seedings, *TaCLE8* and *TaCLE14* are notably expressed whereas *TaCLE2*, *TaCLE13, TaCLE19*, *TaCLE22, TaCLE25, TaCLE26*, *TaCLE27, TaCLE42* and *TaCLE45* are all expressed at low yet measurable levels (**Figure 5**).

In shoot tissue from 1-week-old seedlings, the *TaCLE1, TaCLE5, TaCLE10*, *TaCLE11*, *TaCLE17, TaCLE18, TaCLE20* and *TaCLE41* genes are all expressed (**Figure 4**). Among these, neither *TaCLE18* nor *TaCLE20* is expressed in roots, indicating a potential role in above-ground tissue development. *TaCLE41*, being primarily expressed in young root and shoot tissues reflects potential vascular and root roles like those of its Arabidopsis sister (Ito et al., 2006; Hirakawa et al., 2008; Etchells and Turner, 2010). Further, *TaCLV3, TaCLE2, TaCLE13, TaCLE25, TaCLE26, TaCLE42* and *TaCLE45* are all expressed in 3-week shoot tissue, although the transcript levels of the latter two genes are very low (**Figure 5**). *TaCLV3* expression in shoot tissue suggests a potentially similar role to Arabidopsis *CLV3* in maintaining the shoot apical meristem (Clark et al., 1995; Fletcher, 1999), as its expression is moderate in whole seedling tissue but increased when the tissue is narrowed down to just the shoot while being absent in roots.

Pennycress rosette and rosette leaf tissues also express numerous *CLE* genes. *TaCLE1, TaCLE5, TaCLE7, TaCLE9, TaCLE10*, *TaCLE11*, *TaCLE17, TaCLE18, TaCLE20*, *TaCLE41* and *TaCLE43* transcripts are all detectable in rosette and rosette leaf tissue (**Figure S2**). Additionally, *TaCLE22, TaCLE27* and *TaCLE40* display higher mRNA expression levels in whole seedlings than in either shoots or roots (**Figure 5**), suggesting that all of these genes may function during the vegetative phase.

During the reproductive phase, we detect expression of *TaCLE1, TaCLE5, TaCLE7, TaCLE9, TaCLE10, TaCLE11, TaCLE12* and *TaCLE17* in inflorescences (**Figure S2**; **Figure 4**), as well as *TaCLE18, TaCLE20* and *TaCLE41* in inflorescences and open florescences (**Figure 4**). The same set of genes is expressed in young green siliques, with *TaCLE10, TaCLE11, TaCLE12* and *TaCLE41* showing higher levels of transcription in siliques than in other reproductive tissues.

Seed yield is intrinsically tied with seed weight, as seed weight increases results in an increased harvest index, the seed weight divided by the dry shoot mass. However, little is understood about the role of *CLE* genes in seed development beyond the involvement of *AtCLE8* in embryo and endosperm size regulation (Fiume and Fletcher, 2012). Our transcriptome data indicate that at least 8 *TaCLE* genes are expressed in green seeds: *TaCLE5, TaCLE7, TaCLE10, TaCLE11, TaCLE12, TaCLE17, TaCLE20* and *TaCLE43* (**Figure 4**), excluding *TaCLE14* for which the extremely high level of expression is likely to be an artifact. Interestingly, although *AtCLE5*, *AtCLE7* and *AtCLE12* gene expression is absent in Arabidopsis seed tissue (Klepikova et al., 2016), their pennycress homologs *TaCLE5*, *TaCLE7* and *TaCLE12* are all expressed in green seeds (**Figure 4**). Conversely, *AtCLE17* and *TaCLE17* are both expressed in seed tissue whereas *AtCLE16* transcription is notably absent. The similarity in expression pattern, as well as the greater amino acid similarity in the CLE motif (**Figure 2**), provide further suggestion that the single copy *TaCLE17* gene is more closely related to *AtCLE17* than its sister gene *AtCLE16*. Understanding the functions of *CLE* genes expressed in pennycress seeds could provide a path forward for domestication while also furthering understanding of the family’s role in this crucial step of plant reproduction.

Members of the CLAVATA3/ESR-related peptide family regulate many important biological processes in plants and have played a role in the domestication of diverse crop species. Our study presents the sequence information, conservation, and expression analysis of this family in the emerging biofuel crop pennycress. Using a combination of evolution and transcriptomics analysis, we have provided a solid foundation for future domestication efforts by elucidating potential candidates for future genetic engineering efforts. Such domestication efforts can provide a significant financial incentive that could accelerate the adoption of pennycress as a cover crop to maintain soil health by preventing runoff and erosion during an otherwise fallow planting period. Also, a deeper understanding of this family across different species can provide further insight into the role of *CLE* genes in plant growth and development.

## Data availability statement

The data presented in this study can be found online in the supplemental material at https://www.frontiersin.org/articles/10.3389/fpls.2023.1240342/full#supplementary-material. Genomic data used in this study have previously been deposited in the NCBI Sequence Read Archive under accession number SRP033211 (Dorn et al., 2015). Expression data has previously been deposited in the ENA Sequence Read Archive under accession number PRJEB46635 (Nunn et al., 2022).

## Author contributions

LH and JF conceived and designed the study. LH conducted the experiments and acquired the data. LH and JF analyzed the data. LH wrote the first draft of the manuscript. All authors contributed to the article and approved the submitted version.
