# Supplementary material for: The CLAVATA3/ESR-related peptide family in the biofuel crop pennycress
Source: Front Plant Sci. 2023 Aug 4;14:1240342. doi: 10.3389/fpls.2023.1240342 (PMC10436580; doi:10.3389/fpls.2023.1240342)
Supplement: Supplementary file 1 [file DataSheet_1.pdf]

Supplemental Data for The CLAVATA3/ESR-related (CLE) Peptide Family in the Biofuel Crop Pennycress

Lynne Hagelthorn and Jennifer C. Fletcher

Correspondence to: jfletcher@berkeley.edu

This PDF file contains:

Figure S1

Figure S2

Figure S3

Table S1

Table S2

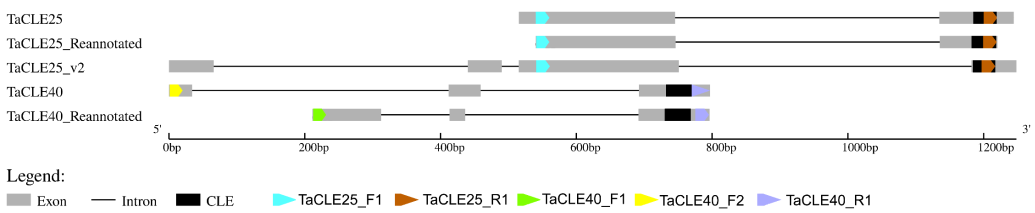
TaCLE25
TaCLE25_Reannotated
TaCLE25_v2
TaCLE40
TaCLE40_Reannotated
5'
3'
0bp
200bp
400bp
600bp
800bp
1000bp
1200bp
Legend:
Exon
Intron
CLE
TaCLE25_F1
TaCLE25_R1
TaCLE40_F1
TaCLE40_F2
TaCLE40_R1

Supplemental Figure 1. Reannotation of the *TaCLE25* and *TaCLE40* loci. Reannotation of genomic locations for *TaCLE25* and *TaCLE40* are shown. In addition, primers that are denoted in Supplementary Table 2 are shown as arrowheads.

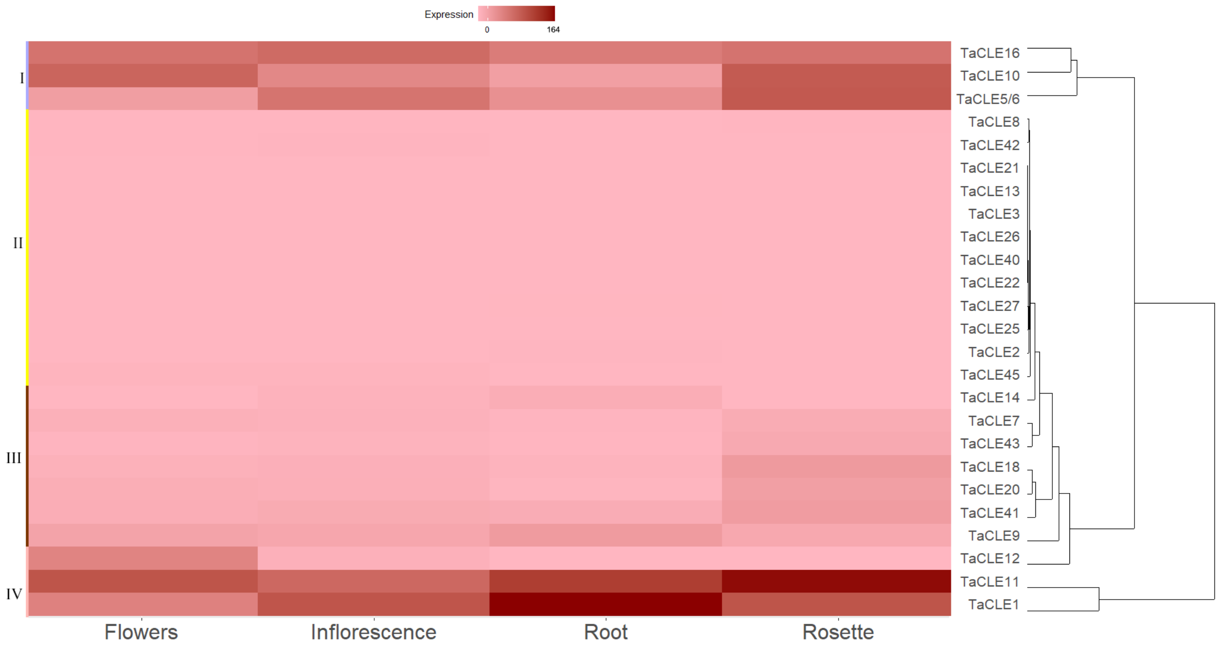
Expression
0
164
I
II
III
IV
TaCLE16
TaCLE10
TaCLE5/6
TaCLE8
TaCLE42
TaCLE21
TaCLE13
TaCLE3
TaCLE26
TaCLE40
TaCLE22
TaCLE27
TaCLE25
TaCLE2
TaCLE45
TaCLE14
TaCLE7
TaCLE43
TaCLE18
TaCLE20
TaCLE41
TaCLE9
TaCLE12
TaCLE11
TaCLE1
Flowers
Inflorescence
Root
Rosette

Supplemental Figure 2. Heatmap of *TaCLE* gene expression in representative aerial and root tissues. Pennycress *CLE* gene expression displays a four-group expression pattern demarcated I-IV.

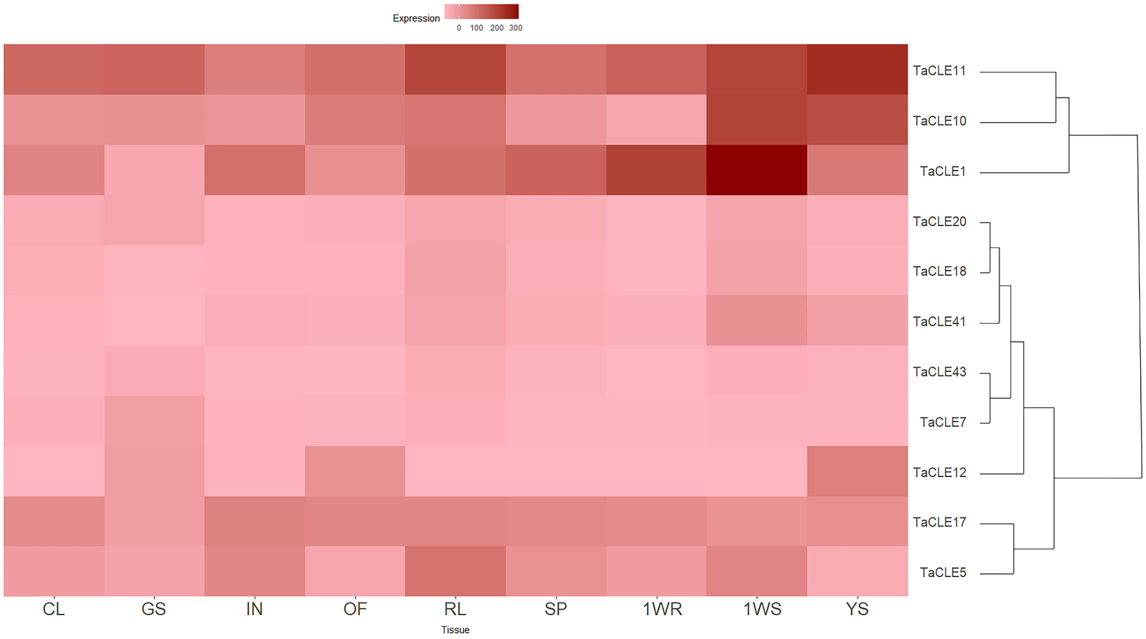

Expression
0 100 200 300
TaCLE11
TaCLE10
TaCLE1
TaCLE20
TaCLE18
TaCLE41
TaCLE43
TaCLE7
TaCLE12
TaCLE17
TaCLE5
CL
GS
IN
OF
RL
SP
1WR
1WS
YS
Tissue

Supplemental Figure 3. Heatmap of *TaCLE* gene expression across nine tissue types. Pennycress *CLE* gene expression levels in nine distinct tissues, with *TaCLE14* omitted due to a potential artefact. CL, cauline leaves; GS, green seeds; IN, inflorescences; OF, open florescences; RL, rosette leaves; SP, seed pods; 1WR, roots from 1-week-old seedlings; 1WS, shoots from 1-week-old seedlings; YS, young green siliques.

# SignalP-6.0	Organism: Eukarya		Time stamp: 20221022011003	
#ID	Prediction	OTHER	SP(Sec/SPI)	CS Position
TaCLE5	NO SP 0.500663		(1499350)	
TaCLE8	NO SP 1.000019		0.000000	
TaCLE12	NO SP 1.000031		0.000000	
TaCLE17	NO SP 0.669173		0.330824	

Supplementary Table 1. SignalP 6.0 predictions for identified peptides with no signal peptide sequence.

Name	Purpose	Sequence 5’ – 3’
TaCLE40 F1 New Annotation	cDNA amplification	ATGGCGGCGATGAGATAC
TaCLE40 F2 Draft Annotation	cDNA amplification	ATGATATTTGTCTGGCTCGTTCC
TaCLE40 R1	cDNA amplification	TATGGAGAAGTAAAGGGAATGTGG
TaCLE19 F1	cDNA amplification	ATGAAGATAAAGGGTTTGATATTGGCTTC
TaCLE19 R1	cDNA amplification	TCACCTGTTGTGAAGTGGATTTGG
TaCLE25 F1	cDNA amplification	ATGGGTGGAAATGGCATTAGAG
TaCLE25 R1	cDNA amplification	TCATACTCGTGGTGGTCGTC
TaCLV3 F1	cDNA amplification	GTTAAGGGAGATGAGAGTTGTTCCTTG
TaCLV3 R1	cDNA amplification	ATGGAATCGAAAAGTCTGGTGC
TaCLE45 F1	qPCR amplification	ATGTTGGGTTCCAGTACAAGAATTATG
TaCLE45 R1	qPCR amplification	TCAAGAGAACGGCTCGGATTTG
TaCLE3 F1	qPCR amplification	ATGGCAAGTTTCAAGTTATGGTTTTG
TaCLE3 R1	qPCR amplification	TTAGTGATGTCTAGGGTCCGGTC
TaCLE2 F1	qPCR amplification	ATGGCTAAGTTAAGCTTTACTCTATGC
TaCLE2 R1	qPCR amplification	CTAATGATGTTGTGGGTCGGG
TaCLE13 F1	qPCR amplification	ATGGCAACGAGTAGAGTCTCAC
TaCLE13 R1	qPCR amplification	TCAATGGTGCAAGGGGTTTG
TaCLE17 F1	qPCR amplification	ATGGGACGTGTTGATGATCAAG
TaCLE17 R1	qPCR amplification	TTAGTTGTGGAGAGGATTGGGACC
TaCLE26 F1	qPCR amplification	ATGCGAAATCTCCGTTCCTTTC
TaCLE26 R	qPCR amplification	CTAGGAGACAGCCTTTTGCGAG
TaCLE21 F1	qPCR amplification	ATGAAACGAGACGTTGTAATCATTG
TaCLE21 R1	qPCR amplification	CTACCTGTTGTGCAAAGGATTTG
TaCLE42 F	qPCR amplification	ATGAGATCTCTTCACATCATCATTTCAC
TaCLE42 R	qPCR amplification	CTACCTATTGGAGATGGGATTTGG
TaCLE8 F	qPCR amplification	TTATGGATTATGTAGAGGATTGGGACC
TaCLE8 R1 v1	qPCR amplification	ATGGCAGTTATTCATGAAGATTCATC
TaCLE22 F	qPCR amplification	ATGGGAAATTACTACTCTAGAAGAAAATCTC
TaCLE22 R	qPCR amplification	TTATCTATTGTGCAAAGGATTAGGACCTG
TaCLE27 F	qPCR amplification	ATGACTCATGCTCGAGAATGG
TaCLE27 R	qPCR amplification	CTAGTTATGGAGAGGATCTGGACAAC
TaCLE14 F1	qPCR amplification	CGCCGGGATTCATTTCTCTTA
TaCLE14 R1	qPCR amplification	CTTCTCTAGCTGAAACTCCA
TaCLE8 R2 v2	Sequence Verification	ATGAAAGAGTTGAAATGTGGTTCTG
TaCLE25 Exon 2 v2 F	Sequence Verification	ATGGATGTTCTGCTCAGTC

Supplementary Table 2. Primers used in this study. A summary of all primers used for amplification and sequencing of cDNA fragments as well as for gene expression analysis using RT-qPCR.
